# Targeting cytotoxic T lymphocytes for cancer immunotherapy

**DOI:** 10.1038/sj.bjc.6602022

**Published:** 2004-07-13

**Authors:** J Maher, E T Davies

**Affiliations:** 1 Cancer Research UK Breast Cancer Biology Group, Division of Cancer Studies, Guy's King's and St Thomas's School of Medicine, Guy's Hospital, St Thomas Street, London SE1 9RT, UK; 2 Department of Clinical Immunology, King's College Hospital, The Rayne Institute, 123 Coldharbour Lane, London SE5 9NU, UK

**Keywords:** CTL, vaccine, adoptive immunotherapy, gene therapy

## Abstract

In light of their preeminent role in cellular immunity, there is considerable interest in targeting of cytotoxic T-lymphocytes to cancer. This review summarises the active and passive immunotherapeutic approaches under development to achieve this goal, emphasising how recent advances in tumour immunology and gene transfer have impacted upon this field.

The challenge of developing cancer immunotherapy is one of the longest standing goals of immunology, dating back to the late 19th century. Despite many setbacks, recent developments have rejuvenated the sense of optimism in this quest. Almost 30 years after their development, monoclonal antibodies are now commonly used in the treatment of selected malignancies. While this represents an important advance, much evidence suggests that harnessing of the cellular immune system might contribute even more effectively to this endeavour. The focus of this minireview is to consider how CD8^+^ cytotoxic T lymphocytes (CTL) might be targeted for cancer immunotherapy.

## CYTOTOXIC T LYMPHOCYTES AS EFFECTORS OF ANTITUMOUR IMMUNITY

Cytotoxic T lymphocytes represent a crucial component of the adaptive immune system with particular importance in the control of intracellular pathogens. Effector CTL have the capacity to promote the apoptotic death of carefully chosen target cells, using a combination of granule (perforin/granzyme)- and receptor (Fas/tumour necrosis factor)-mediated mechanisms. While natural killer cells also promote cell death, CTL are distinguished by their exquisite specificity for antigen, which they recognise using a clonally unique T-cell receptor (TCR). Target cells are ‘flagged’ for the attention of CTL when they present antigen-derived peptide fragments on the cell surface, inserted into the groove of class I major histocompatibility (MHC) molecules.

In addition to cytolytic function, a number of other properties render CTL attractive as mediators of antitumour immunity. First, the widespread expression of MHC class I molecules means that CTL could, in principle, be deployed against malignancies of diverse origin. Second, CTL (like all lymphocytes) continuously recirculate throughout the body to seek out antigen, a useful property for the treatment of systemic disease. Third, target recognition is impressively sensitive – even a single peptide–MHC class I complex may trigger cytolysis by a high-avidity effector CTL (Sykulev *et al*, 1996). Finally, CTL also employ nonlytic effector mechanisms including the production of interferon gamma – a cytokine with several direct and indirect antitumour properties (Qin *et al*, 2003).

In light of their lethal payload, generation of effector CTL is tightly regulated and depends upon the antigen-driven differentiation of naïve CD8^+^ T cells. This process is initiated when dendritic cells (DC) take up antigen within peripheral tissues and migrate to the regional lymph nodes. Here, DC are ideally placed to interact with naïve T lymphocytes, which preferentially recirculate through secondary lymphoid tissue. In order to achieve optimum CTL priming, DC must be in an activated (‘licensed’) state. This is generally achieved when DC present processed antigen to MHC class II-restricted CD4^+^ (helper) T cells – the latter then upregulate CD40 ligand that engages DC-associated CD40. Alternatively, DC may be licensed in response to some proinflammatory cytokines or bacterial products that engage Toll-like receptors (Lanzavecchia, 1998).

To achieve optimum expansion of CTL, two additional requirements should be met. First, since CD4^+^ and CD8^+^ T cells are restricted by different MHC families, it is desirable that DC process antigen to enable presentation of derived epitopes both by MHC class I and II. Second, appropriate costimulatory signals must be provided (‘signal 2’) to complement that delivered by the TCR (‘signal 1’). These accessory signals are delivered by several ligands, many of which are upregulated on licensed DC. The best characterised of these are the members of the B7 family, which engage T-cell-associated CD28. Under some circumstances, the resultant clonal expansion of CTL is quite remarkable. In the context of some acute viral infections, CTL of a single specificity may account for more than 40% of peripheral blood CD8^+^ T cells (Callan *et al*, 1998).

## IMMUNE TOLERANCE AND CANCER

**Figure 1:**
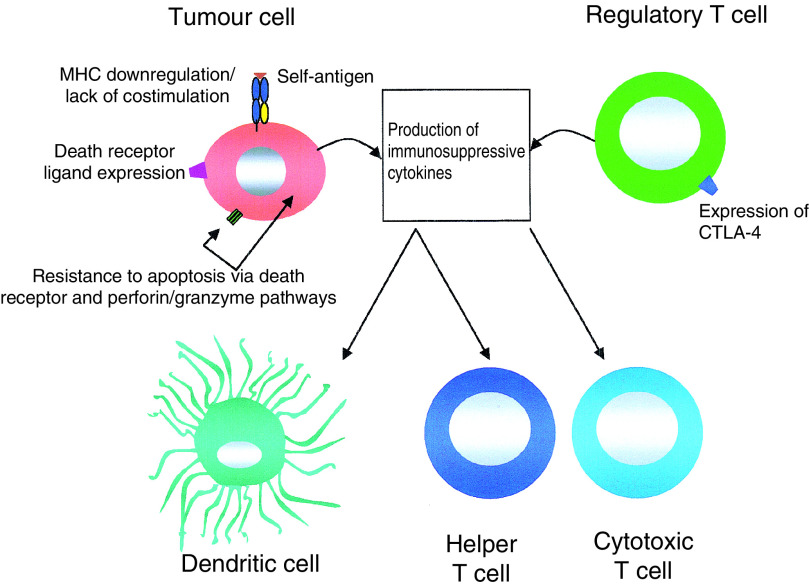
Tumours can escape from immune targeting by CTL in several ways. First, since cancer cells are closely related to ‘self’, they contain a predominance of poorly immunogenic self-antigen. Second, cancer cells have poor antigen-presenting properties since they provide limited costimulation and may downregulate MHC antigen expression. Third, the tumour microenvironment is frequently rich in cytokines that compromise DC differentiation (e.g. interleukin (IL)-6, IL-10, macrophage colony-stimulating factor, vascular endothelial growth factor) or activation, proliferation and effector function of T lymphocytes (e.g. transforming growth factor-*β*). Fourth, cancer cells may acquire resistance to apoptosis, a key effector mechanism of CTL and other cells of the immune system. Of related significance, cancer cells may express death receptor ligands (e.g. Fas ligand) that could facilitate the elimination of tumour-specific T cells (‘immune counterattack’). Finally, it has been suggested that regulatory T-cell subsets (for example, CD4^+^ CD25^+^ Treg cells) promote immune tolerance to cancer. These cells also produce immunosuppressive cytokines and express CTLA-4, a molecule with a number of tolerance-promoting activities.

To counteract the attention of CTL and other effector arms of the immune system, cancer cells deploy several immune evasion strategies (summarised in Figure 1

). Many of these diversionary tactics rely upon the ability of cancer cells to harness systems that maintain immune tolerance to self. Immune tolerance is mediated in part by deletion of autoreactive lymphocytes, although this would be difficult to reverse using an immunotherapeutic approach. By contrast, nondeletional tolerance mechanisms may in principle prove more amenable to therapeutic manipulation. For example, increasing evidence has implicated a number of regulatory T-cell subsets in the maintenance of immune tolerance both to self and to derived malignancies. These include CD4^+^ CD25^+^ Treg cells, IL-10-producing Tr1 cells, transforming growth factor (TGF)-*β*-producing Th3 cells and CD8^+^ regulatory T-cell subsets (Nelson, 2004). In light of this, it is conceivable that the depletion or suppression of such cellular populations might provide a useful adjunct in the immunotherapy of malignant disease.

## VACCINATION STRATEGIES THAT TARGET CTL TO TUMOUR ANTIGENS

In principle, CTL may be targeted to cancer using one of two approaches, based upon the classical establishment of active or passive immunity. The greatest success of clinical immunology has been the development of vaccines for the prophylaxis of infectious disease. Indeed, some such vaccines are proving useful in the prevention of virus-related malignant disorders, notably hepatoma (Chang *et al*, 1997). The majority of infectious disease vaccines achieve host immunity since they elicit a protective antibody response. By contrast, most tumour vaccines have focused upon the induction of tumour-reactive CTL.

**Figure 2:**
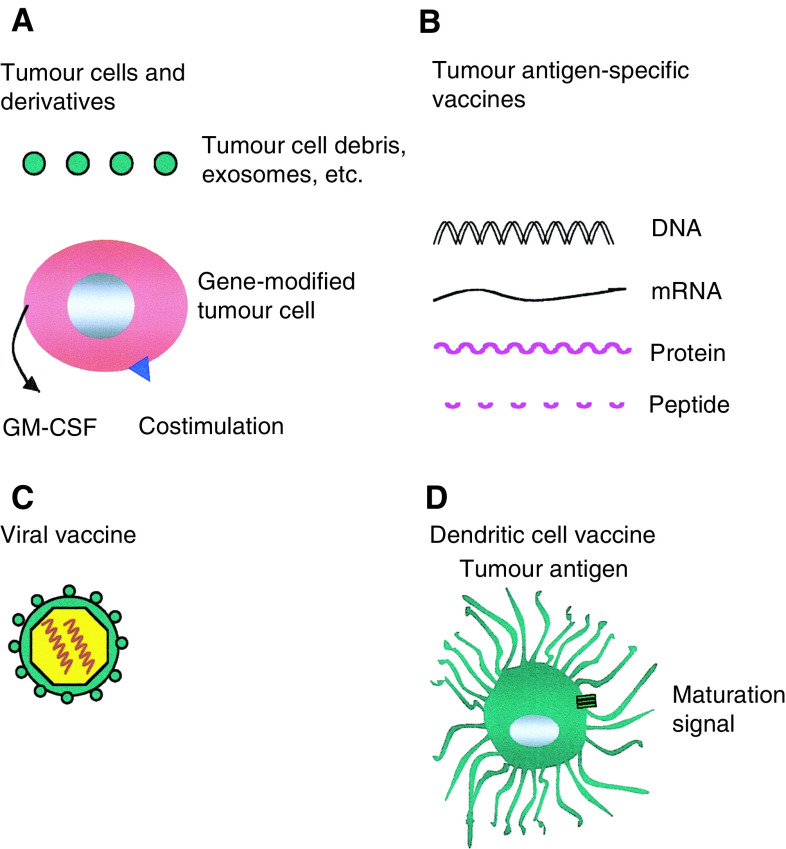
To break immune tolerance against cancer, several vaccine-based strategies are under development. (**A**) To target a broad spectrum of antigens, tumour cells may be engineered to secrete immunomodulatory cytokines, such as GM-CSF, and/or costimulatory ligands (e.g. B7). Alternatively, whole tumour cell derivatives may be administered together with an appropriate adjuvant and/or delivery system. (**B**) Vaccines based upon discrete tumour antigen(s) may be formulated in several ways – for example, as naked DNA, protein or derived peptide(s). Since many tumour antigen-derived peptides bind weakly to MHC, sequence alterations may be incorporated to increase binding affinity (heteroclitic approach). A variety of targeting motifs may be fused with such molecular vaccines in an attempt to direct delivery (for example to dendritic cells) and/or preferential processing by the MHC class I or class II pathway. (**C**) Viral delivery systems are powerfully immunogenic, particularly when live (attenuated) vectors are used. These agents show particular promise in ‘prime-boost’ strategies, in which antigen is administered sequentially by distinct means. (**D**) Dendritic cells are potent antigen-presenting cells that may be used to deliver tumour antigen in several forms. Whereas immature DC may promote tolerance, mature DC are highly immunogenic and may even break tolerance to self-antigen.

One traditional approach to the development of a cancer vaccine involves the use of inactivated whole tumour cells (or derived extracts – Figure 2A

). More recently, this approach has been extended with the development of tumour cell-based vaccines engineered to express immunomodulatory cytokines and/or costimulatory ligands (e.g. following genetic modification or fusion with DC). An important difficulty with the use of such complex vaccines is the fact that relevant antigens are often obscure and thus *in vitro* immune monitoring may prove difficult.

In recent years, the scientific rationale underlying tumour vaccination has been strengthened enormously by the demonstration that nonviral malignancies express tumour antigens. This crucial advance, pioneered by Thierry Boon and co-workers (van der Bruggen *et al*, 2002), has resulted in the development of vaccines based upon defined molecular targets. In the majority of cases, such antigens are targets for CTL and derive from molecules that are overexpressed in tumour cells, compared to their normal counterparts. The recognition of the close relationship between tumour antigens and ‘self’ provides a molecular explanation for one of the key obstacles to the breaking of immune tolerance to cancer. In light of this difficulty, a very large number of antigen-specific vaccines are under development (Figure 2B–C), based upon peptides, protein, mRNA or DNA expressed from plasmid or viral vectors (Whelan *et al*, 2003). To enhance immunogenicity, a range of adjuvants have been tested many of which promote DC activation. Indeed, an increasing area of study involves the use of DC-based vaccines engineered to express antigen (Figure 2D; Cerundolo *et al*, 2004). Many tumour vaccines have successfully expanded CTL responses to appropriate antigens (Coulie and van der Bruggen, 2003). Furthermore, some promising studies have been reported in experimental animal models, particularly in the setting of protection from tumour challenge (Morris *et al*, 2003).

Recently, several small clinical studies of therapeutic vaccination have been performed. In general, these trials have confirmed the safety of the approach and have sometimes demonstrated induction of CTL and/or antibody responses to vaccine components (e.g. Knutson *et al*, 2001). These findings suggest that tumours bearing defined antigens can be targeted immunotherapeutically. Somewhat disappointingly however, apart from sporadic responses in isolated individuals, clinical outcomes have generally been disappointing (Finn, 2003; Morris *et al*, 2003).

What lessons have been learned that might improve the effectiveness of tumour vaccination? A key point illustrated by many animal studies is the relative ineffectiveness of this approach in the setting of advanced tumour burden. Consequently, it may prove much more fruitful to test suitable vaccines in patients with minimal residual disease. A further option is to combine vaccination with other therapeutic modalities, as recently illustrated in acute promyelocytic leukaemia (Padua *et al*, 2003). Third, it may be worth developing broadly applicable vaccines that simultaneously target CTL to multiple tumour-associated epitopes (Graff-Dubois *et al*, 2002). In parallel, it is important to determine why therapeutic vaccination is poorly effective in advanced disease and to understand those instances where significant responses are observed. A variety of new techniques permit the monitoring of antigen-specific CTL responses, including ELISPOT, tetramer analysis and intracellular cytokine detection (Clay *et al*, 2001). While studies to date have only occasionally correlated results of such assays with clinical responses, this is likely to provide a fertile area for further research.

## ADOPTIVE IMMUNOTHERAPY USING *EX VIVO* GENERATED CTL

Passive (adoptive) immunotherapeutic approaches also represent an attractive means to target CTL to tumour cells. In some *in vivo* experimental models, adoptive immunotherapy using amplified CTL has demonstrated greater success than vaccination approaches that target the same tumour type (Romieu *et al*, 1998). Adoptive immunotherapy generally involves the administration of large numbers of T cells, thereby bypassing tolerance mechanisms that limit the activation and expansion of CTL. In clinical practice, this approach is best illustrated by treatment of some haematological malignancies with allogeneic stem cell transfer, or donor leucocyte infusion. In this setting, there is compelling evidence that CTL and other effector cells deliver a ‘graft *vs* leukaemia effect’ that contributes importantly to therapeutic efficacy. Transferred CTL are also of established benefit in the treatment of some virus-related malignancies, such as Epstein–Barr virus (EBV)-related post-transplant lymphoproliferative disease (Rooney *et al*, 2001). However, evidence that such cellular therapies can achieve meaningful control of common solid tumours is more limited. Furthermore, it is well known that allogeneic T cells can mediate graft *vs* host disease, with potentially lethal consequences. Consequently, there is a need to broaden the applicability and enhance the safety of this approach, preferably with the use of tumour-specific autologous T lymphocytes.

The development of adoptive T-cell immunotherapy for solid tumours has been pioneered by Steven Rosenberg and co-workers. Following the demonstration that IL-2 could achieve responses in a small number of patients with malignant melanoma, this group subsequently developed techniques to expand tumour-infiltrating lymphocytes (TIL) *in vitro.* When TIL were infused into patients, a modest improvement in response rate became apparent, although success was hampered by poor *in vivo* persistence of transferred cells (Rosenberg *et al*, 1994). Over the ensuing years, it has emerged that TIL are enriched for MHC class I-restricted CTL with specificity for known melanoma antigens including MART-1 and gp100. More recently, this group have shown that following lymphodepletion, TIL undergo impressive IL-2-driven expansion *in vivo*, increasing the clinical response rate significantly (Dudley *et al*, 2002).

These studies elegantly provide proof of principle for the clinical potential of adoptive immunotherapy. To translate this approach more widely, systems are required to achieve the rapid *ex vivo* expansion of CTL targeted to relevant tumour antigens. Fortunately, a number of strategies are under development that may achieve this.

## GENETIC APPROACHES TO CTL-BASED ADOPTIVE IMMUNOTHERAPY

A recent key development has been the application of gene transfer-based strategies to target CTL to cancer cells. Several avenues of investigation are under development at this time. One promising approach involves the use of ‘artificial antigen-presenting cells’ that permit the *in vitro* expansion of tumour-specific CTL (Latouche and Sadelain, 2000). To achieve this, NIH3T3 fibroblasts have been genetically engineered to express a chosen peptide epitope, together with an MHC class I molecule, *β*2 microglobulin and a series of costimulatory ligands. Using this system, impressive expansion of CTL of the desired specificity can be achieved. To further boost *in vitro* CTL expansion, many groups have demonstrated the potent growth-promoting effect of IL-15 (e.g. Brentjens *et al*, 2003).

**Figure 3:**
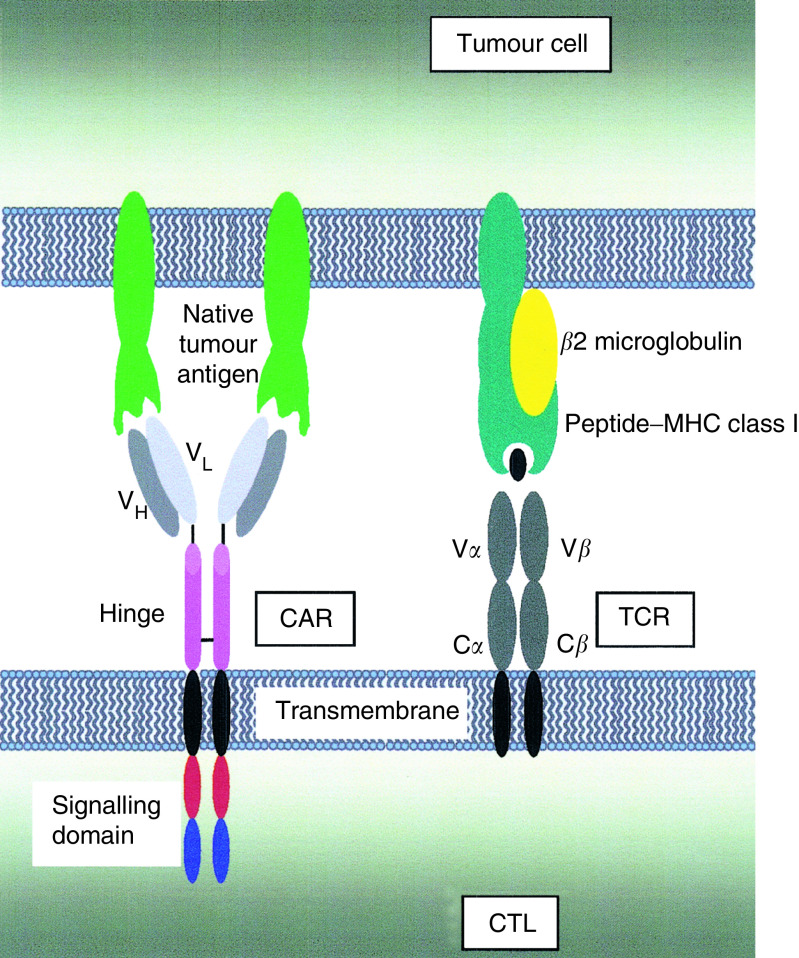
Antigen recognition by chimeric antigen receptors (CAR) is most commonly mediated by a single chain antibody fragment, comprising a variable heavy (VH) and variable light (VL) chain domain, joined with a short linker (left). In recent CAR designs, the intracellular domain comprises a modular array of fused signalling elements that together provide a TCR-like and a costimulatory signal. By contrast, the TCR recognises processed antigen, presented as a peptide–MHC complex (right). This in turn triggers signal transduction by the CD3 complex (not shown).

An alternative approach is to genetically modify T cells or CTL, thereby retargeting specificity to a chosen tumour antigen (Eshhar, 1997). Most commonly, this is achieved using a ‘chimeric antigen receptor’ (CAR) in which an antibody-derived single chain fragment (scFv) is coupled via a hinge to an appropriate signalling element (Figure 3

). Chimeric antigen receptors are generally expressed on the T-cell surface as a single gene-encoded homodimer, enabling the MHC-independent recognition of native tumour-associated antigen. Several such fusion receptors have been constructed with specificity for molecules expressed by solid and haematological malignancies (e.g. Eshhar, 1997, Gong *et al*, 1999).

In most early CAR designs, the signalling domain was chosen to deliver a surrogate TCR-like stimulus. The CD3*ζ* cytoplasmic domain delivers a potent signal 1 and is well suited for this purpose. Despite fears about TCR signalling in cancer-bearing hosts, it is reassuring that CD3*ζ*-based CAR retain potent activity when expressed in T lymphocytes derived from such patients (Gong *et al*, 1999; Brentjens *et al*, 2003; Sheen *et al*, 2003).

Most tumours do not provide adequate T-cell costimulation. Consequently, there has been considerable interest in the development of CAR that provides such accessory signals. An important initial development was the construction of scFv/CD28 fusion receptors (Alvarez-Vallina and Hawkins, 1996). More recently, CAR have been designed in which the signalling domains of CD3*ζ* and CD28 have been fused in series, creating molecules that can deliver both a functional signal 1 and signal 2 (Finney *et al*, 1998). When such ‘second-generation’ CAR are expressed in human T cells, they can be repeatedly activated *in vitro* by coculture with antigen-expressing tumour cells. Each cycle of stimulation results in rapid death of the tumour targets, followed by IL-2-driven proliferation of CAR-grafted T cells (Maher *et al*, 2002). More recently, it has been shown that several alternative costimulatory domains can be fused in series with CD3*ζ* to create CAR with distinct functional properties (Finney *et al*, 2004). An alternative approach to promote sustained survival of CAR-grafted CTL involves selective gene-transfer into EBV-responsive T cells, which are known to persist for prolonged periods *in vivo* (Rossig *et al*, 2002). In parallel to these developments, CAR technology has received an important boost with the demonstration that this approach can achieve sustained control of an established *in vivo* tumour burden (Haynes *et al*, 2002; Brentjens *et al*, 2003). Significantly, those studies have also emphasised the importance of costimulation.

An alternative genetic strategy involves the introduction of a new TCR with specificity for a defined, tumour-associated peptide–MHC complex. A number of *in vitro* studies have successfully used this system to redirect the antigenic specificity of both CD4^+^ and CD8^+^ T cells. This approach is attractive in that it allows access to a greater repertoire of protein antigens than CAR, since the latter requires that the targeted antigen is expressed on the cell surface. Furthermore, it might be anticipated that ectopic TCR would prove less immunogenic than CAR that originate from (nonhumanised) rodent hybridoma-based scFv. However, these advantages are balanced by some important disadvantages. Since the TCR is a heterodimer, this strategy requires the regulated coexpression of two gene products (TCR *α* and *β* chains). In principle, such exogenous receptor subunits may heterodimerise with endogenous TCR subunits, generating complexes with autoreactive potential. This potential difficulty may be overcome by inclusion of sequences that only permit dimerisation of the ectopic TCR subunits. A second disadvantage is the frequent downregulation of MHC class I molecules observed in cancer, providing an opportunity for immune escape from the genetically modified CTL (Gilboa, 1999). Thirdly, owing to the highly polymorphic nature of the human MHC (human leucocyte antigen – HLA) system, ectopic TCR would need to be matched to the HLA haplotype of the patient, imposing additional logistical constraints.

An important consideration for all forms of immunotherapy is the tumour microenvironment, which is frequently poorly conducive to CTL function (Figure 1). Many malignancies are associated with overproduction of immunosuppressive cytokines such as TGF-*β*, although genetic approaches may be used to circumvent this (Gorelik and Flavell, 2001). Alternatively, the microenvironment may be decorated with a suitable immunopotentiating agent, such as the tumour-necrosis factor superfamily member, LIGHT. Recently, this has been reported to achieve a dramatic recruitment of naïve CTL to malignant deposits, where they are primed and elicit impressive antitumour immunity (Yu *et al*, 2004).

The exciting opportunities afforded by genetic modification of T cells have set the stage for a number of clinical studies that are currently underway. However, a cautionary note is warranted in light of the development of leukaemia in two children following treatment of X-linked severe combined immunodeficiency using gene-modified haemopoietic stem cells. A full discussion of this complex issue is beyond the scope of the current review (see Kohn *et al*, 2003). It is clear, however, that safety must be paramount in the clinical testing of such novel treatments. Consequently, it may be appropriate to include ‘suicide genes’ in adoptively transferred T cells to allow their elimination when required. The best studied of these approaches involves expression of the herpes simplex virus thymidine kinase gene, which renders T cells susceptible to ganciclovir.

## CONCLUSIONS

Recent developments mean that immunotherapy is likely to play an increasingly important role in cancer therapy. Adoptive immunotherapy may find its niche in the debulking of disease that is resistant to conventional therapeutic regimens. By contrast, vaccination may prove most useful in the context of lower tumour burden and as a means to attain longer-term immunity and memory. In both settings, CTL are likely to play a key role, justifying the widespread interest in targeting of these key immune effector cells to cancer.
